# Differences in the corrective effects of vertical transposition accompanied by recession–resection of the horizontal rectus muscles for complicated vertical deviation

**DOI:** 10.1038/s41433-024-03270-3

**Published:** 2024-07-26

**Authors:** Shin-ya Nakao, Manabu Miyata, Akinari Yamamoto, Kentaro Kawai, Kenji Suda, Eri Nakano, Miho Tagawa, Akitaka Tsujikawa

**Affiliations:** 1 Department of Ophthalmology, Kyoto University Graduate School of Medicine, Kyoto, Japan; 2 Department of Ophthalmology, Osaka Red Cross Hospital, Osaka, Japan

**Keywords:** Eye diseases, Predictive markers

## Abstract

**Background/objectives:**

To investigate whether the corrective effect differs between upward and downward transpositions or between exotropia and esotropia in vertical transposition accompanied by horizontal rectus muscle recession–resection.

**Subjects/methods:**

This prospective study investigated 41 patients with concomitant exotropia or esotropia with small-angle vertical deviation who underwent unilateral vertical transposition accompanied by horizontal rectus muscle recession–resection and were followed up for 1 year postoperatively. We analysed the vertical deviation corrective effect, defined as the corrective amount per displacement distance (°/tendon width [TW]). We compared the corrective effects between upward and downward transpositions and between exotropia and esotropia. Additionally, we investigated the correlation between the corrective effect and the studied parameters.

**Results:**

The 1-year vertical corrective effect was 5.2 ± 4.6° (9.0 ± 8.1 prism dioptres [Δ])/TW. The 1-year vertical corrective effect of upward transposition (7.9 ± 4.0° [13.8 ± 7.0Δ]/TW) was higher than that of the downward transposition (3.9 ± 4.4° [6.8 ± 7.7Δ]/TW, *P* = 0.009). In contrast, upward and downward transposition did not differ between exotropia and esotropia (*P* = 0.62). Multivariate analyses revealed that the 1-year vertical corrective effect correlated with the vertical transposition direction (upward or downward) and preoperative vertical deviation but did not correlate with the disease type (exotropia or esotropia). The 1-year motor success (vertical deviation ≤ 5Δ) rate was 89%.

**Conclusion:**

The vertical corrective effect of vertical transposition accompanied by horizontal rectus muscle recession–resection is greater in upward transposition than in downward transposition; however, it does not differ between exotropia and esotropia.

## Introduction

Patients with horizontal strabismus often also exhibit vertical deviations. Previous studies have reported that 50% of patients with constant esotropia and 25% of patients with heterotropia experience small-angle concomitant hypertropia of up to 3 prism dioptres (Δ) [1, 2]. Vertical deviation is more troublesome than horizontal deviation because the vertical fusion range is smaller than the horizontal range. However, performing recession–resection for horizontal strabismus and manipulating the superior or inferior rectus muscles to correct the vertical deviation can increase operation time, anterior ischaemia risk, and burden on patients [3, 4].

**Fig. 1:**
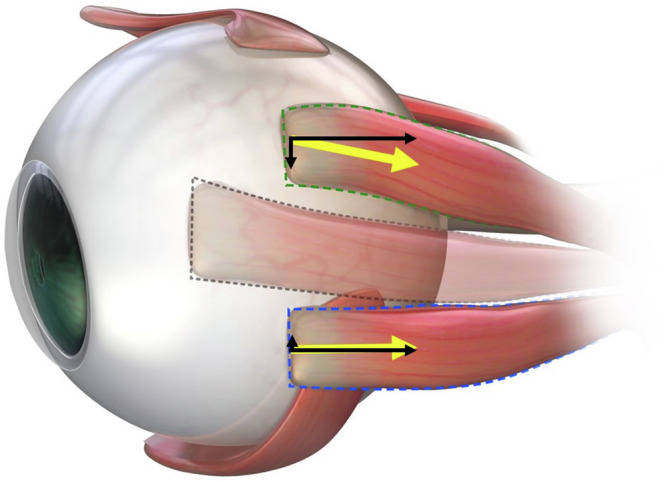
Schema to demonstrate our hypothesis. As the horizontal rectus muscle is displaced inferiorly in older persons, muscle strength may be greater with upward transposition than with downward transposition. Furthermore, because the downward transposition of the lateral rectus muscle moves it closer to the insertion of the inferior oblique muscle, adhesions can occur quickly around the insertion site, which may affect the surgical outcomes. However, the lateral rectus muscle remains far from the oblique muscles after upward transposition.

Foster and Pemberton first reported the effectiveness of vertical transposition accompanied by horizontal rectus muscle recession–resection to correct not only the horizontal but also vertical deviation [5]. Subsequently, several studies have demonstrated its effectiveness [2, 6–9]. However, the corrective effect was variable, ranging from 0.5 to 2.0Δ/millimetre (mm), and the predictive factors are yet to be determined. A magnetic resonance imaging study reported that the horizontal rectus muscles are displaced inferiorly in older participants relative to the centre of the globe and are accompanied by a presumably inferior location of the corresponding orbital pulleys [10]. Therefore, we hypothesised that the corrective effect would differ between upward and downward transpositions (Fig. 1).

This study aimed to investigate whether the corrective effect of vertical transposition differs between upward and downward transpositions or between disease types (exotropia and esotropia) and to identify the predictors of the corrective effect of vertical transposition in horizontal strabismus surgery for horizontal strabismus with vertical deviation.

## Subjects and methods

The Kyoto University Graduate School of Medicine Ethics Committee approved this prospective cohort study (Kyoto, Japan, Approval Number: R0134). All study protocols adhered to the tenets of the Declaration of Helsinki. All participants provided verbal informed consent. The participants’ records and information were anonymised before analysis.

### Participants

We prospectively recruited consecutive patients with concomitant exotropia or esotropia (excluding A- or V-pattern strabismus) with vertical deviation between May 2015 and December 2021 from the Department of Ophthalmology and Visual Sciences at the Kyoto University Graduate School of Medicine. The inclusion criteria were unilateral recession–resection with vertical transposition of the horizontal rectus muscles performed by one surgeon (MM) and follow-up for 1-year post-surgery. In this study, we included only cases in which we transposed the medial and lateral rectus muscles vertically in the same direction in equal amounts, and we excluded cases with different amounts or directions of vertical transposition.

Before surgery, all patients underwent a comprehensive examination, including measurement of the ocular position at 5 metres (m) and 0.3 m using the prism and alternate cover test (PACT), measurement of best-corrected visual acuity (BCVA) using a Landolt chart, estimation of binocularity (binocular single vision [BSV], suppression, or diplopia) using the Bagolini-striated glasses test, and measurement of stereopsis using the TNO stereo test (Laméris Ootech BV, Nieuwegein, Netherlands). We defined cases in which vertical deviation of 2–18Δ at a far distance was to be included. We routinely measured the axial length in most patients preoperatively (IOL Master 500, Carl Zeiss Meditec, Dublin, CA, USA). The same surgical amount should have a different ratio to the size of the eye; therefore, we consider that axial length might affect the surgery’s corrective effect. We used the axial length of the operated eye for the analysis. At the 3-month and 1-year time points post-surgery, we measured the ocular position and estimated binocularity in all patients. The exclusion criteria were using an adjustable suture (because we considered that minor errors in the surgical amount compared to fixed sutures could affect the results), history of past strabismus surgery, incomitant strabismus (including cranial nerve palsy, dissociated vertical deviation, and progressive myopic strabismus), poor BCVA of <20/40 in either eye or a difference of >10Δ in horizontal or vertical deviation while assessing the ocular position at a far distance between two examiners (one doctor and one certified orthoptist). The same doctor performed measurements preoperatively and postoperatively. Different certified orthoptists examined the patients in some cases. However, the examination instruments and conditions were identical.

### Surgical procedure

One surgeon (MM) performed the procedure as described in a previous report [11]. Briefly, we made a limbal conjunctival incision and 3–4 mm radial incisions after administering eye drops containing 4% lidocaine and 1% lidocaine with 0.001% epinephrine. We administered SubTenon’s injections of 2% lidocaine at the near sides of the two horizontal muscles. We performed recession–resection with upward or downward transposition by fixation with two and three knots of 7-0 nylon (7-0 Ortho suture, Handaya, Tokyo, Japan), respectively. We did not perform hang-back recession using absorbable suture; instead, fixed suture was performed using non-absorbable suture, as a previous report had suggested that there was greater return with absorbable suture compared to non-absorbable suture and a higher strabismus recurrence rate [12]. We performed vertical transposition in equal amounts using downward transposition for hyperdeviation and upward transposition for hypodeviation of the operated eye. We moved the suture position after vertical transposition vertically parallel to the original muscle insertion site by the targeted surgical amount from the original muscle insertion site in the resection procedure or from the location where we moved the targeted surgical amount horizontally from the original muscle insertion site in the recession procedure. The surgeon measured the distance between the limbus and centre of the muscle insertion along with tendon width (TW) at the level of the insertion in 0.1 mm steps using a Castroviejo calliper and confirmed the distance between the two tips of the calliper with a ruler because the calliper’s scale is sometimes inaccurate. We used the mean muscle insertion distance and width for the analyses. Finally, we closed the conjunctival wound with 8-0 silk (Mani Inc., Tochigi, Japan).

### Target surgical amount

We determined the target surgical amount by considering not only the results of the PACT but also those of the prism adaptation test (PAT) [13, 14]. Before surgery, we lent a Fresnel prism with an appropriate prism dioptre (and glasses with plano lenses if the patient had no glasses) based on the PAT results at the outpatient clinic and instructed the patient to wear the Fresnel prism glasses for at least 30 min daily for 1 month. We determined the required surgical amount of horizontal recession–resection using the horizontal deviation at 5 m based on our institutional surgery table and by referring to Park’s table [15]. We calculated the amount of vertical transposition as a correction of 10Δ/1 TW (Δ/TW), and increased or decreased it according to the amount of vertical deviation to avoid overcorrection [7]. Concretely, for a vertical deviation of 2Δ, the surgical amount was 1/5 TW, for 3Δ, 1/4 TW, for 4Δ, 1/3 TW, for 5Δ–6Δ, 1/2 TW, for 7Δ–9Δ, 2/3 TW, and for 10Δ, 1 TW. The maximum TW transposition done in this study was 1 TW. If the preoperative vertical deviation was >10Δ, we performed 1 TW vertical transposition. We used TW as a unit for the vertical transposition distance because we confirmed the TW in the surgery and decided on the fixed sites. When the vertical deviation differed between 5 and 0.3 m, we selected the smaller deviation as the target.

### Analyses of the corrective amount and effect

We converted Δ of ocular deviation at 5-m fixation to degrees (°) for analyses because Δ would lead to inaccurate statistical analyses or calculations. The corrective amount (°) was the value of the change in the ocular position from preoperative to 3 months and 1 year postoperatively (+, improved; −, worsened). We calculated the horizontal surgery amount as the sum of the amounts of the medial and lateral rectus muscles. At the same time, we determined the vertical surgery amount as the amount of equal transposition of the medial and lateral rectus muscles themselves. We determined the horizontal surgery amount in mm and the vertical surgical amount by the TW at muscle insertion; thus, we calculated the corrective effects, defined as the corrective amount per displacement distance, of the horizontal and vertical deviations as °/mm and °/ TW, respectively.

### Motor and sensory success

We defined motor success as a postoperative vertical deviation at a far distance of ≤5Δ (~2.9°) among patients with preoperative vertical deviation >5Δ, regardless of the reversal of vertical deviation, in accordance with a previous report [16]. We defined sensory success as postoperative BSV, which was determined using the Bagolini-striated glasses test among patients without preoperative BSV. We defined sensory failure as suppression or diplopia. We did not set the definition of surgical failure and did not use reoperation as an exclusion criterion for surgical success.

### Statistical analysis

We presented all data as the mean ± standard deviation (the median and interquartile range shown in the tables). We investigated the correlation between the corrective effect and studied parameters using Spearman’s correlation coefficient. We used multivariate linear regression analysis to examine the parameters correlated with the 3-month or 1-year vertical corrective effect, respectively; therefore, we did not include both the 3-month and 1-year corrective effects in one multivariable analysis. In the multivariable analysis, we used only data at far distance. Studied parameters with *P* < 0.10 in Spearman’s correlation analyses were included as the independent variables. We compared the differences in the sex and disease type between the upward and downward transposition surgeries using the chi-square test. We performed the Mann–Whitney *U* test to compare the corrective effects and parameters between the upward and downward transposition surgeries and between exotropia and esotropia. We used analysis of variance with repeated measures and post hoc analysis with Bonferroni correction to compare the mean strabismus angles at preoperative, 3-month, and 1-year postoperative points. We used Cochran’s *Q* test to compare the rates of motor and sensory success. We performed all statistical analyses using SPSS software (version 27.0; IBM, Armonk, NY, USA). We set statistical significance at *P* < 0.05.

## Results

### Patient characteristics

Overall, 65 patients met the inclusion criteria; of these, 24 satisfied the exclusion criteria (1 with an adjustable suture, 11 with a history of strabismus surgery, 4 with a poor VA, 5 with cranial nerve palsy, 1 with progressive esotropia, and 2 with an inter-investigator difference >10Δ). In total, we investigated 41 participants (age, 48.5 ± 26.6 years; 18 women) (Table 1); all patients had concomitant strabismus, 26 had exotropia (13 with intermittent exotropia and 13 with constant exotropia), and 15 had esotropia (14 with congenital esotropia and 1 with acute acquired esotropia). We operated on 2 patients aged below 12 years at the time of surgery under general anaesthesia, while the other 39 received topical anaesthesia. The mean muscle width and mean distance from the limbus were 9.5 ± 0.9 mm and 5.4 ± 0.5 mm, respectively.

**Table 1:** Patient characteristics (*n* = 41).

Preoperative parameters	Age, years	48.5 ± 26.6
	Sex (men/women), *n* (%)	23 (56.1)/18 (43.9)
	Disease (XT/ET), *n* (%)	26 (63.4)/15 (36.6)
	Binocular single vision present, *n* (%)	12 (29.3)
	LogMAR BCVA	−0.08 ± 0.13
	Axial length, mm	24.65 ± 1.60^a^
Intraoperative parameters	Tendon width, mm	9.5 ± 0.9
	Distance between the insertion and limbs, mm	5.4 ± 0.5
	Sum of recession and resection amount, mm	12.7 ± 2.8
	Vertical surgical amount, TW	0.5 ± 0.3

Data are presented as means ± standard deviation where applicable.

*XT* exotropia, *ET* esotropia, *logMAR BCVA* logarithm of the minimal angle of resolution best-corrected visual acuity, *TW* tendon width.

The data for ^a^ was missing in six eyes.

### Changes in the deviation and corrective effect

The preoperative horizontal and vertical deviations at 5-m fixation were 21.6 ± 7.0° (39.6 ± 12.3Δ) and 3.2 ± 2.1° (5.6 ± 3.7Δ), respectively (Table 2). The preoperative, 3-month and 1-year horizontal deviations at 0.3 m fixation were 23.3 ± 9.9° (43.1 ± 17.5Δ), 5.1 ± 4.7° (8.9 ± 8.2Δ), and 6.5 ± 5.2° (11.4 ± 9.1Δ), whereas the vertical deviations were 2.8 ± 2.3° (4.9 ± 4.0Δ), 0.8 ± 0.9° (1.4 ± 1.6Δ), and 0.8 ± 1.1° (1.4 ± 1.9Δ), respectively. The horizontal and vertical deviations at 3 months and 1 year postoperatively significantly improved (*P* < 0.001 in both). In this study, no patient required reoperation during the 1-year follow-up. Post hoc analyses revealed that the 3-month and 1-year horizontal deviation, as well as the 3-month and 1-year vertical deviation, were significantly smaller than the preoperative values (all *P* < 0.001). Vertical deviation at far distance did not differ between 3 months and 1 year postoperatively, indicating no regression of the surgical effect (*P* = 0.87). The vertical corrective effect at far distance was 5.4 ± 4.1° (9.5 ± 7.1Δ)/TW for 3 months and 5.2 ± 4.6° (9.0 ± 8.1Δ)/TW for 1 year postoperatively. The vertical corrective effect at a near distance was 4.7 ± 3.1° (8.3 ± 5.5Δ)/TW for 3 months and 5.1 ± 3.6° (9.0 ± 6.2Δ)/TW for 1 year postoperatively.

**Table 2:** Changes in deviation and corrective effect.

		Preoperative	3 months	1 year
Horizontal	Deviation, degree	21.6 ± 7.0 (19.3 [16.7–25.4])	3.2 ± 3.4 (2.3 [0.9–4.6])	4.1 ± 4.2 (2.3 [1.1–5.7])
	Corrective amount, degree	–	19.8 ± 7.5 (18.7 [13.8–23.7])	18.8 ± 7.9 (18.7 [13.3–22.6])
	Corrective effects, degree/mm	–	3.1 ± 0.9 (3.1 [2.5–3.5])	2.9 ± 1.0 (2.9 [2.4–3.5])
Vertical	Deviation, degree	3.2 ± 2.1 (2.9 [1.7–4.0])	1.0 ± 1.0 (0.6 [0.0–1.7])	1.1 ± 1.2 (0.6 [0.0–1.7])
	Corrective amount, degree	–	2.8 ± 2.5 (2.3 [1.1–4.0])	2.8 ± 2.8 (1.7 [1.1–4.0])
	Corrective effect, degree/TW	–	5.4 ± 4.1 (5.2 [2.6–8.0])	5.2 ± 4.6 (5.2 [3.4–8.6])

Data are expressed as mean ± standard deviation. Values inside brackets are presented as medians (interquartile range, 25th–75th percentile). Strabismus angles and associated values were calculated and shown in absolute degrees.

*TW* tendon width.

### Correlation analysis of the vertical corrective effect

Univariable analyses revealed that the 3-month and 1-year vertical corrective effects at far distance correlated with the vertical transposition direction and preoperative vertical deviation (Table 3). In multivariable analyses, the 3-month and 1-year vertical corrective effects at far distance correlated with the direction of vertical transposition and preoperative vertical deviation, but did not correlate with the disease type.

**Table 3:** Correlation between the 3-month or 1-year vertical correction effect and the studied parameters.

		3 months				1 year			
		Univariable analysis		Multivariable analysis		Univariable analysis		Multivariable analysis	
		*P*	*r*	*P*	*β*	*P*	*r*	*P*	*β*
Preoperative parameters	Age	0.54	−0.10	–	–	0.80	−0.04	–	–
	Sex (1, male; 2, female)	0.24	0.19	–	–	0.69	0.06	–	–
	Axial length	0.26	0.20	–	–	0.28	0.19	–	–
	Disease (1, XT; 2, ET)	0.70	0.06	–	–	0.61	0.08	–	–
	Horizontal deviation	0.97	0.01	–	–	0.64	−0.08	–	–
	Vertical deviation	<0.001*	0.46	0.01*	0.40	<0.001*	0.53	<0.001*	0.43
Intraoperative parameters	Horizontal surgical amount	0.82	0.04	–	–	0.56	0.09	–	–
	Transposition direction (1, up; 2, down)	0.01*	−0.41	0.03*	−0.31	0.01*	−0.41	0.04*	−0.29
	Transposition amount	0.59	0.09	–	–	0.35	0.15	–	–
	Tendon width	0.97	0.01	–	–	0.80	−0.04	–	–
	Distance from limbs	0.80	−0.04	–	–	0.95	0.01	–	–

*XT* exotropia, *ET* esotropia.

*Statistically significant (*P* < 0.05).

### Differences between upward and downward transposition and between exotropia and esotropia

The 3-month and 1-year corrective effects of upward transposition at far distance were 7.9 ± 4.2° (13.9 ± 7.4Δ)/TW and 7.9 ± 4.0° (13.8 ± 7.0Δ)/TW, respectively. These values were higher than those for downward transposition (3-month, 4.3 ± 3.5° [7.5 ± 6.1Δ]/TW, *P* = 0.008; 1-year, 3.9 ± 4.4° [6.8 ± 7.7Δ]/TW, *P* = 0.009) (Table 4). However, we observed no significant differences in any of the other parameters, including age (*P* = 0.10), vertical transposition amount (*P* = 0.46), axial length (*P* = 0.22), preoperative vertical deviation (*P* = 0.40), muscle width (*P* = 0.59), distance from the limbus (*P* = 0.11), and disease type (*P* = 0.87).

**Table 4:** Differences in the studied parameters between upward and downward transposition.

		Upward	Downward	*P* value
Patients, *n*		13	28	–
Age, years		58.1 ± 25.8	44.1 ± 26.3	0.10
Women, *n* (%)		7 (54)	11 (39)	0.38^a^
Disease (XT/ET), *n*		8/5	18/10	0.87^a^
Tendon width at the insertion, mm		9.7 ± 0.6 (9.7 [9.4–9.8])	9.4 ± 1.0 (9.6 [9.1–9.9])	0.59
Distance between the insertion and limbus, mm		5.6 ± 0.4 (5.5 [5.5–5.9])	5.3 ± 0.5 (5.3 [4.9–5.8])	0.11
Transposition amount, TW		0.5 ± 0.3 (0.3 [0.3–0.7])	0.5 ± 0.3 (0.5 [0.3–0.5])	0.45
Vertical deviation, degree	Preoperative	4.0 ± 2.9 (3.4 [1.7–5.7])	2.8 ± 1.5 (2.6 [1.7–3.6])	0.40
	3-month	0.7 ± 0.5 (0.6 [0.6–1.1])	1.1 ± 1.1 (0.9 [0.0–1.9])	0.54
	1-year	0.8 ± 1.0 (0.6 [0.0–1.1])	1.2 ± 1.3 (1.0 [0.0–2.3])	0.46
Corrective amount, degree	3-month	3.8 ± 2.9 (3.4 [2.3–5.2])	2.3 ± 2.1 (1.7 [1.1–3.4])	0.08
	1-year	4.0 ± 3.2 (2.3 [1.7–6.3])	2.3 ± 2.5 (1.7 [1.1–3.4])	0.17
Corrective effect, degree/TW	3-month	7.9 ± 4.2 (7.7 [5.7–10.3])	4.3 ± 3.5 (3.4 [2.3–6.9])	0.009*
	1-year	7.9 ± 4.0 (6.9 [5.2–9.6])	3.9 ± 4.4 (4.6 [2.3–6.0])	0.01*

Data are presented as mean ± standard deviation where applicable. Values inside brackets are presented as median (interquartile range, 25th–75th percentile).

*TW* tendon width.

*Statistically significant (*P* < 0.05).

^a^Chi-square test; for the others, the Mann–Whitney *U* test was used.

The 3-month and 1-year corrective effects of exotropia at far distance (3-month, 5.5 ± 4.3° [9.5 ± 7.5Δ]/TW; 1-year, 5.1 ± 4.7°[8.9 ± 8.2Δ]/TW) were not significantly different from those of esotropia (3-month, 5.4 ± 3.7° [9.5 ± 6.5Δ]/TW, *P* = 0.70; 1-year, 5.3 ± 4.7° [9.2 ± 8.1Δ]/TW, *P* = 0.62), although we observed significant differences in the preoperative vertical deviation at far distance (*P* = 0.002) and vertical transposition amount (*P* = 0.03). However, other parameters, including age (*P* = 0.70), axial length (*P* = 0.38), muscle width (*P* = 0.40), and distance from the limbs (*P* = 0.20), showed no significant differences between the exotropia and esotropia groups.

### Motor and sensory success

Among 19 patients with preoperative vertical deviation >5Δ, 19 (100%) and 17 patients (89%) achieved motor success 3 months and 1 year after surgery, respectively. They demonstrated significant improvement (*P* < 0.001 in both) (Supplementary Table). We achieved sensory success at both the 3-month and 1-year time points after surgery in 23 patients (79%) among 29 patients without preoperative BSV. They also showed a significant improvement in the success rate (*P* < 0.001).

## Discussion

This study showed that vertical transposition with horizontal rectus muscle recession–resection for horizontal strabismus with vertical deviations was effective in reducing the vertical deviations and demonstrated a higher motor success rate (3 months, 100%; 1 year, 89%), compared to that in a previous report [2]. We found that the predictors of vertical transposition were preoperative vertical deviation and transposition direction (upward or downward) but not age or the disease type (exotropia or esotropia). Furthermore, we showed that the 3-month and 1-year upward transposition corrective effects were significantly higher than those of downward transposition. Our findings suggest that vertical transposition is effective but requires attention to preoperative vertical deviation and transposition direction.

As expected, the corrective effect differed between upward and downward transpositions, whereas all other studied parameters, including the disease type and vertical deviation, did not. To the best of our knowledge, there have been no previous reports on this difference. A previous study reported variability in the vertical corrective effects of vertical transposition [7]; however, the reason for this difference remains unclear. We considered that the tension was stronger in upward transposition because the horizontal rectus muscles were displaced inferiorly with ageing [10], consequently, the upward transposition corrective effect was stronger than the downward transposition corrective effect. However, the current study found no correlation between the corrective effect and age (*P* = 0.54). Another possibility is a difference in the anatomical insertion sites of the superior and inferior oblique muscles. The insertion site of the inferior oblique is close to the lateral rectus muscle, while the insertion site of the superior oblique is distant from the lateral rectus muscle. A recession with downward transposition of the lateral rectus muscle moves the insertion of the lateral rectus close to the insertion of the inferior oblique muscles. Adhesions around the insertions may affect the corrective effect. Further research is required in this area.

As we observed no significant difference in the ocular position and corrective effect between far and near distances; we used only data from far distances in the multivariable analysis. Multivariable correlation analyses revealed that the vertical corrective effect correlated with the transposition direction and preoperative vertical deviation. The correlation between the corrective effect and preoperative vertical deviation was positive. Hence, a more significant preoperative vertical deviation would have a stronger corrective effect. We determined the surgical amount (transposition distance from the origin) based on our institutional surgical table, with a correction of 10Δ/1 TW. However, our correlation analysis results suggest that the relationship between the surgical and corrective amounts is not linear (Supplementary Figure). The variation in the corrective effect might be reduced if the surgeon determines the surgical amount with this point in mind, as well as the difference in the transposition direction.

The motor success rate in the present study was relatively higher than that in a previous report (63%). However, there were some differences, including the definition of motor success (≤one-half of the preoperative vertical angle or <8Δ) and surgical table (fixed as 4-mm transposition) [2]. In that study, the motor success rate of superior or inferior muscle recession combined with horizontal rectus muscle surgery was 71%, which was lower than that in the present study. We believe that the higher success rate in the present study was mainly because our surgical table was in accordance with the preoperative vertical deviation.

Considering that 1 TW was measured as 9.5 mm intraoperatively in the present study, we can convert the 3-month and 1-year corrective effects of 5.4 and 5.2°/TW both to 1.0Δ/mm. Based on these results, we considered the vertical corrective effects in this study to be similar to those previously reported as 0.9Δ/mm [7]. However, these mean values include upward and downward transpositions. Our findings suggest that we should divide the surgical table into upward and downward transpositions.

This study has some limitations. First, the sample size was small. We require further extensive cohort studies (e.g. multi-centre studies) to clarify the validity of our findings. Second, we did not compare the outcomes of horizontal rectus muscle recession–resection with and without vertical transposition. Simple recession–resection may have a corrective effect on vertical transposition. A previous report suggested that hypertropia associated with intermittent exotropia (range, 5–14Δ at a distance) was resolved by horizontal rectus muscle surgery without vertical transposition for intermittent exotropia [17]. However, the patients in this study were <18 years old (range, 2–18 years), and the strabismus examination may have been inaccurate; therefore, simply comparing these results with those of the current study may be controversial. Third, we did not classify exotropia and esotropia according to binocularity (phoria or tropia). A previous study reported more vertical overcorrection in intermittent exotropia than in non-intermittent exotropia [2]. However, we could not assign our small sample to many subgroups for further statistical analyses. Fourth, although no patient complained of torsional diplopia in this study, we did not routinely measure the postoperative torsional deviation. Therefore, we could not assess the effect of vertical transposition on torsion.

In conclusion, convenient vertical transposition accompanied by recession–resection for horizontal strabismus improved the vertical deviation with a high surgical success rate. Determining the vertical transposition amount based on our findings that the vertical corrective effect of upward transposition was higher than that of downward transposition and that the vertical corrective effect correlated with preoperative vertical deviation may lead to better surgical outcomes.

## Summary

### What was known before

- Vertical transposition accompanied by recession–resection of the horizontal rectus muscles has been reported to be effective in correcting vertical deviation. However, the corrective effect is variable and the predictive factors have not been determined yet.

### What this study adds

- This study showed that the vertical corrective effect of vertical transposition accompanied by recession–resection of the horizontal rectus muscles was greater in upward transposition than in downward transposition; however, it did not differ between exotropia and esotropia.

## Supplementary information

Supplementary Table

Supplementary Figure

## Data Availability

All data generated or analysed during this study are included in this published article and its Supplementary Information files.
