# Supplementary material for: Differences in the corrective effects of vertical transposition accompanied by recession–resection of the horizontal rectus muscles for complicated vertical deviation
Source: Eye (Lond). 2024 Jul 26;38(17):3252–7. doi: 10.1038/s41433-024-03270-3 (PMC11584644; doi:10.1038/s41433-024-03270-3)
Supplement: Supplementary file 1 — Supplementary Table [file 41433_2024_3270_MOESM1_ESM.docx]

| **Supplementary Table. Motor and sensory success in vertical deviation** | | |
| --- | --- | --- |
|  | 3-month | 1-year |
| Motor success (n = 19), n (%) | 19 (100) | 17 (89) |
| Sensory success (n = 29), n (%) | 23 (79) | 23 (79) |
