# Supplementary material for: Differences in the corrective effects of vertical transposition accompanied by recession–resection of the horizontal rectus muscles for complicated vertical deviation
Source: Eye (Lond). 2024 Jul 26;38(17):3252–7. doi: 10.1038/s41433-024-03270-3 (PMC11584644; doi:10.1038/s41433-024-03270-3)
Supplement: Supplementary file 2 — Supplementary Figure [file 41433_2024_3270_MOESM2_ESM.docx]

Supplementary Figure. Scatter plots of the surgical and corrective amount and quadratic approximations.

Scatter plots for the association between surgical amount and corrective amount at 3 months (A) and 1 year (B) postoperatively. The horizontal axis represents the surgical amount (tendon width), and the vertical axis represents the corrective amount (°). The black dots represent the mean values of the corrective amount in each surgical amount. Vertical bars represent the standard errors. We analysed the approximate equations in linear, logarithmic, quadratic, cubic, and exponential functions, among which the quadratic approximation with a *P* value of <0.05 and the highest R^2^ value is indicated by a solid line. We have also indicated the approximate linear line by a dotted line. The linear approximation had an R^2^ of 0.88 and *P* value of 0.05, while the quadratic approximation had an R^2^ of 0.95 and *P* value of 0.01 at 3 months postoperatively. The linear approximation had an R^2^ of 0.95 and *P* value of 0.001, while the quadratic approximation had an R^2^ of 0.98 and *P* value of 0.004 at 1 year postoperatively. These approximations show that the relationship between the surgical and corrective amount is better suited to a quadratic approximation than a linear one.
